# Alteration of Gene Expression Profile in Kidney of Spontaneously Hypertensive Rats Treated with Protein Hydrolysate of Blue Mussel (*Mytilus edulis*) by DNA Microarray Analysis

**DOI:** 10.1371/journal.pone.0142016

**Published:** 2015-10-30

**Authors:** Junli Feng, Zhiyuan Dai, Yanping Zhang, Lu Meng, Jian Ye, Xuting Ma

**Affiliations:** 1 Institute of Seafood, Zhejiang Gongshang University, Hangzhou, China; 2 School of Biological and Chemical Engineering, Zhejiang University of Science and Technology, Hangzhou, China; University of Florida, UNITED STATES

## Abstract

Marine organisms are rich sources of bioactive components, which are often reported to have antihypertensive effects. However, the underlying mechanisms have yet to be fully identified. The aim of this study was to investigate the antihypertensive effect of enzymatic hydrolysis of blue mussel protein (HBMP) in rats. Peptides with *in vitro* ACE inhibitory activity were purified from HBMP by ultrafiltration, gel filtration chromatography and reversed-phase high performance liquid chromatography. And the amino acid sequences of isolated peptides were estimated to be Val-Trp, Leu-Gly-Trp, and Met-Val-Trp-Thr. To study its *in vivo* action, spontaneously hypertensive rats (SHRs) were orally administration with high- or low-dose of HBMP for 28 days. Major components of the renin-angiotensin (RAS) system in serum of SHRs from different groups were analyzed, and gene expression profiling were performed in the kidney of SHRs, using the Whole Rat Genome Oligonucleotide Microarray. Results indicated although genes involved in RAS system were not significantly altered, those related to blood coagulation system, cytokine and growth factor, and fatty acids metabolism were remarkablely changed. Several genes which were seldom reported to be implicated in pathogenesis of hypertension also showed significant expression alterations after oral administration of HBMP. These data provided valuable information for our understanding of the molecular mechanisms that underlie the potential antihypertensive activities of HBMP, and will contribute towards increased value-added utilization of blue mussel protein.

## Introduction

Hypertension is a global health concern, thought to affect up to 30% of the adult population in developed and developing countries [1]. It plays a causative role in the onset of stroke, myocardial infarction, heart failure, peripheral arterial disease, and chronic kidney disease. Essential hypertension, the most common type of hypertension and to which 90–95% of cases belong, is manifested as an increase in an individual's blood pressure (BP) due to unknown causes [1]. According to the expert, this class of hypertension can be improved with lifestyle modification by paying attention to physical activity and the kind of foods consumed, and reducing the level of stress [2].

It is well known the renin-angiotensin (RAS) system is the primary pathway for regulating BP and vascular tone [3]. The RAS pathway is initiated in the kidney by the conversion of prorenin zymogen to the active form, renin. Renin then cleaves angiotensinogen (Ang) to release angiotensin I (Ang I). Ang I circulates in the blood until its C-terminal dipeptide residue is cleaved by angiotensin I-converting enzyme (ACE, EC 3.4.15.1) to form angiotensin II (Ang II), which acts as a potent vasoconstrictor. Ang II binds its cell surface receptors to trigger the secretion of aldosterone from adrenal glands, leading to increased reabsorption of salt and water and elevation of BP by arterial constriction [4,5]. Due to its special role in BP regulation, ACE has long been investigated as a major physiological target for developing antihypertensive drugs. However, these ACE inhibiting drugs appear to pose many side effects that discourage their use by hypertensive patients [6]. Thus, ACE inhibiting natural products have been vigorously pursued during the last two decades as agents for lowering BP. For example, the BIOPEP database currently documents (as of June 2013) 3211 bioactive peptide sequences and 684 of them are ACE inhibitors (BIOPEP database, http://www.uwm.edu.pl/biochemia).

Based on the current literature, physiological antihypertensive effects of bioactive peptides are often attributed to ACE inhibition. However, emerging evidences indicated that ACE inhibition might not be completely responsible for attenuating hypertension [6,7]. In fact, antihypertensive effects of bioactive components could be mediated via multiple mechanisms such as sugar and lipid metabolism, endothelial function, response to oxygen stress, and ion channels. For instance, amaranth trypsin-digested glutelins could induce nitric oxide production in coronary endothelial cells, and corresponding nitric oxide-induced vasodilation in isolated rat aortic rings [8]. Tryptophan-containing dipeptides (Trp-His, His-Trp,Trp-Leu and Trp-Val) produced vasodilation effect in isolated rat thoracic aortic rings, as they could inhibit intracellular Ca^2+^ increase in vascular smooth muscle cells [9].

Marine organisms are rich sources of bioactive compounds. Among them, fish and shellfish proteins are structurally diversified and highly balanced with amino acid content, thus they become the particularly interested substrates to produce peptides with multifunctional bioactivities [10]. A number of studies reported the antihypertensive potential of protein hydrolysate produced from oyster, catfish, tuna, salmon, sardine, alaskan pollack, rainbow trout, giant jellyfish, sea cucumbers, shrimp and bonito [11–17]. Several of them are regarded as traditional food in East Asian countries to treat asthma and hypertension [15,17].

In previous studies, Wang et al. [18] reported the purification and characterization of an antioxidant peptide derived from blue mussel (*Mytilus edulis*) protein hydrolysate. Je et al. [19] reported the isolation of an ACE inhibitory peptide (Glu-Val-Met-Ala-Gly-Asn-Leu-Tyr-Pro-Gly) from the sauce of fermented blue mussel, with IC_50_ value of 19.34 mg/ml *in vitro*. The antihypertensive effect of this peptide was also evaluated in spontaneously hypertensive rats (SHRs) following oral administration, and BP significantly decreased after peptide ingestion. These results suggested that blue mussel proteins may possess sequences with potentially BP lowering activity. However, antihypertensive peptides derived from enzymatic hydrolysis of blue mussel protein (HBMP) have not yet been studied. Therefore, this study was carried out to investigate the antihypertensive effect of HBMP in SHRs, an established animal model of essential hypertension. Several ACE inhibitory peptides were purified from the hydrolysate, and their amino acid sequences were studied. Furthermore, to elucidate its underlying mechanism, DNA microarray analyses were performed to obtain gene expression profiles in kidney tissue of SHRs after repeated oral administration of high- or low-dose of HBMP. The results showed that expression of 1621 unique genes (*p* ≤ 0.05) were changed (fold change ≥ 1.5) in SHRs at a dose of 20 mg/kg/day when compared with control rats, whereas the expression of 1568 unique genes were changed (fold change ≥ 1.5) at a dose of 10 mg/kg/day. Among them, genes involved in RAS system and those linked to vascular inflammation, blood clotting system, cytokines and growth factors were especially studied. The aim of this study was to identify target genes which expression were markedly changed after oral administration of HBMP in an established animal model of essential hypertension, and to understand the molecular mechanism of *in vivo* antihypertensive activities of HBMP.

## Materials and Methods

### Preparation of Protein Hydrolysate of Blue Mussel

Blue mussel (*M*. *edulis*) was purchased from a local shellfish market in October 2014 (Hangzhou, China). Muscle of blue mussel was separated manually and pounded to homogenate. The homogenate was defatted with 95% ethanol (1:4, w/v) at 50°C for 1 h, and then the supernatant was drained. This defatted procedure was repeated for 3 times, and finally the precipitate was freeze-dried (LGJ-1 Freeze-Dryer, Shanghai, China) and stored at -20°C.

Defatted precipitate powder (100 g) was dissolved in 2000 ml distilled water (5% w/v) and hydrolyzed for 4 h using 2.5 g alcalase, which was purchased from Novozymes Biotechnology Co., Ltd. (Tianjin, China), at pH 9.0, 60°C. After 4 h, the digest was heated in boiling water bath for 10 min to inactive enzyme activity. The hydrolysate was centrifuged at 12,000 g for 15 min, supernatants were freeze-dried and stored at -20°C until the time for testing, and named as HBMP.

Chemical analyses of raw muscle and HBMP were performed as follows: protein content was measured by the method of Kjeldahl, using 6.25 as the conversion ratio of nitrogen to crude protein; moisture was measured as the decrease in weight after heating; fat was measured by Soxhlet extraction with petroleum ethe was used as organic solvent; ash content was measured using the direct ashing method; polysaccharide content was studied by phenol-sulfuric acid method with a Multiskan spectrum microplate spectrophotometer (Thermo Fisher Scientific, Waltham, Massachusetts, USA); amino acids content of HBMP was determined quantitatively by High-speed amino acid analyzer L-8800 (Hitachi High-Technologies, Tokyo, Japan).

### Assay for *In vitro* ACE Inhibitory Activity

*In vitro* ACE inhibition assay was performed by measuring the end product, hippuric acid, after an enzymatic reaction between ACE (Sigma A6778) and the substrate hippuryl-histidyl-leucine (HHL) (Sigma H1635). Freeze-dried HBMP samples were dissolved in distilled water to a series of concentration. Then, a sample of 20 μL was pre-incubated with 50 μL substrate (2 .17 mmol/L HHL in 100 mmol/L sodium borate buffer, pH 8.3) at 37°C for 10 min. Addition of 10 μL (2 mU) ACE started the enzymatic reaction that was carried out on a shaker at 37°C for 30 min. The reaction was stopped by adding 85 μL 0.1% trifluoroacetic acid (TFA). The end product, hippuric acid, was then measured by quantitative HPLC (Waters 2996; Waters Scientific Co, MA, USA) on a ZORBAX Eclipse C18 (4.6 mm×210 mm, 5 μm particle size, Agilent Ltd., USA). Isocratic elution was performed with 30% acetonitrile containing 0.1% TFA, at a flow rate of 0.8 mL/min. The effluent was monitored with an ultraviolet detector at 228 nm and the ACE inhibitory activity was calculated as peak area. Five concentrations of each sample were performed for the IC_50_ determination. Each concentration was repeated three times, and the amount of sample needed to inhibit 50% ACE activity was defined as the IC_50_ value.

### Isolation and Analysis of the ACE Inhibitory Peptides from HBMP

The BNH solution was firstly fractionated according to molecular weights (MW) using molecular weight cut-off membranes (Milipore Co, MA, USA) of 10 and 3 kDa at 0.14 MPa, 25°C. Three peptide fractions were obtained according to their MW ranges: >10 kDa, 3–10 kDa, and <3 kDa, respectively. The fractions were concentrated and lyophilised, and *in vitro* ACE inhibitory activity were measured. Then, 300 mg low MW peptide fraction (<3 kDa) was dissolved in 3 mL distilled water and purified by BioLogic DuoFlow medium-pressure liquid chromatography system (Bio-Rad Laboratories, CA, USA), using Sephadex G-15 gel filtration column (2.6× 80 cm, Bio-Rad). The column was eluted with distilled water at a flow rate of 1 mL/min. The elution solution was collected every 1.5 mL and five subfractions were collected and lyophilised. The subfraction with the greatest *in vitro* ACE inhibitory activity was further separated by reversed-phase high performance liquid chromatography (RP-HPLC, Waters 2996; Waters Scientific Co, MA, USA) on a Atlantis T3 column (column size: 4.6×250 mm, 5 μm particle size, Waters Scientific), using a linear gradient of acetonitrile from 5 to 80% containing 0.05% TFA. Finally, the isolated peaks with the greatest potency were selected for peptide identification using a UPLC-MS system, which was conducted on an ACQUITY UPLC (Waters Scientific) interfaced with an API 4000 Q-TRAP (Applied Biosystems, CA, USA).

### SHRs and Measurement of BP

SHRs (8–10 week-old, male, SPF, 280–330 g body weight) were purchased from SLRC Laboratory Animal Inc. (Shanghai, China). SHRs were housed individually in steel cages with controlled room temperature (23 ± 1°C), humidity (55 ± 5%), and lighting (lights on from 06:00 to 18:00). They were fed with standard laboratory diet, and tap water was freely available.

The rats were acclimatized in the above conditions for about a week before the experiment. All the experiments were conducted in accordance with the National Institutes of Health Guide for the Care and Use of Laboratory Animals (NIH publication 85–23, revised 1996), and the experimental protocols were approved by the Ethics Committee on Animal Experimentation of Zhejiang Academy of Medical Sciences. SHRs were randomly divided into three groups (n = 10): control group, rats were orally administered with water (about 3 mL) for 4 weeks; low-dose group, rats were orally administered with HBMP (10 mg/kg/day, about 3 mL) for 4 weeks; high-dose group, rats were orally administered with HBMP (20 mg/kg/day, about 3 mL) for 4 weeks. The HBMP was orally administered at 9:00 every morning, and the BP of each rat was measured at 11:00 on 0, 4, 8, 12, 16, 20, 24 and 28 day, by tail-cuff method with a tail measurement device (ALC-NIBP system, Shanghai Alcott Biotech Co., Ltd., Shanghai, China). Ten BP readings were obtained for each rat and averaged.

### Isolation of Animal Tissue and Biochemical Assays in Serum

At 28 days, all rats were killed with an overdose of pentobarbital anesthesia, and 5 mL blood samples were collected from the heart. These blood samples were transferred immediately into aseptic capped tubes, stood for 30 min, and centrifuged at 1200 g for 20 min. The plasma supernatant was collected and stored at -20°C until further analysis. The kidney was dissected from each rat and immediately stored in liquid nitrogen. Four kidney samples were selected for DNA microarray analyses from each group, by excluding the rats with the highest and lowest changes in BP and body weight.

The levels of Ang, ACE, AngII, endothelial nitric oxide synthase (eNos), high density lipoprotein cholesterol (HDL-C) and low density lipoprotein cholesterol (LDL-C) in serum of SHRs from different groups were separately determined by the enzyme-linked immunoassay (ELISA) or the enzymatic colorimetric method, using kits purchased from NanJing Jian Cheng Bioengineering Institute (Nanjing, China).

### DNA Microarray Analysis

The following procedures for sample preparation and microarray analysis were done at LC Sciences (Houston, TX, USA). Total RNA was extracted and purified from kidney tissue separately, equivalent amounts of RNA from each sample were mixed, and named control group, high-dose group and low-dose group, respectively. Then, the Cy3-labeled cRNA was transcribed from 200 ng of RNA of each group using the Agilent Low Input Quick Amp Labeling Kit (Agilent Technologies, Santa Clara, CA, USA). Cy3-labeled cRNA was hybridized to the Whole Rat Genome Oligonucleotide Microarray ver. 3.0 (4×44k, G2519F-028282) (Agilent Technologies), following the manufacturer's hybridization protocol. After the washing step, the microarray slides were scanned using GenePix 4000B Scanner (Molecular Device, Sunnyvale, CA, USA) according to the manufacture's protocol. Microarray expression data were analyzed using Feature Extraction software (GenePix^®^ Pro 7, Axon Instruments Inc, Foster City, CA) with the default settings for all parameters. The raw data were firstly normalized with the quantile algorithm, and the probes that at least one out of all samples had fluorescence signals in detection were chosen for further analyses. Differentially expressed genes were identified through fold changes as well as *p* value calculated with Student's t-test. The threshold set for up- and down-regulated genes was a fold change ≥ 1.5 with a *p* value ≤ 0.05.

For those genes which were differently expressed among groups, gene ontology (GO) and KEGG pathway analyses were performed using DAVID software. GO covers three domains; 1. cellular component, the parts of a cell or its extracellular environment; 2. molecular function, the elemental activities of a gene product at the molecular level and 3. biological process, operations or sets of molecular events with a defined beginning and end, pertinent to the functioning of integrated living units: cells, tissues, organs, and organisms. KEGG pathway analysis was used to assess which pathways were over-represented in a given set of genes. This allowed for identification of pathways significantly affected after oral administration of HBMP.

### Reverse Transcription-Quantitative Real-Time Polymerase Chain Reaction (qRT-PCR)

To confirm the accuracy of the microarray results, the same RNA samples were subjected to qRT-PCR to determine the mRNA levels of *Ang*, *ACE*, *angiotensin II type-1 receptor* (*AT-1*), *prostaglandin-endoperoxide synthase 1* (*COX-1*), *adrenoceptor beta 3* (*AR-β3*), *interleukin 24* (*IL-24*) and *peroxisome proliferator-activated receptor δ* (*Pparδ*). These genes were randomly selected, and the *β-actin* was used as internal reference gene. The cDNA was synthesized from 200 ng of the total RNA using PrimeScript 1st strand cDNA synthesis kit (Takara, Dalian, China). qRT-PCR analysis was performed with a LightCycler (Roche Diagnostics, Mannheim, Germany) using the LightCycler TaqMan Master mix (Roche Diagnostics), with the forward and reverse primers listed in S1 Table. Thermal cycling was carried out under the following condition: 95°C for 2 min, followed by 40 cycles of 10 s at 95°C, and 30 s at 60°C. Fluorescence data were analyzed with LightCycler software (Roche Diagnostics). After the reaction, the threshold cycle (*C*_*t*_) was determined using default threshold settings, and the comparative *C*_*t*_ method (2^-ΔΔCt^) was used to determine the relative abundance of each gene in control or HBMP-administrated rats.

### Statistical Analysis

Body weight, BP data, and concentration of biochemical parameters in serum of SHRs are expressed as means with standard errors (SE). Statistical comparisons between groups were performed using one-way analysis of variance (ANOVA, SPSS 13.0 software) followed by Student's t-test, and differences are considered significant when *p* < 0.05. All microarray data were analyzed using the Bonferroni correction and Fisher exact test, and the average value and real-time PCR from 3 RNA samples are presented as the mean ± SE. Differentially expressed genes were identified through fold changes as well as *p* value calculated with Student's t-test. The threshold set for up- and down-regulated genes was a fold change ≥ 1.5 with a *p* value ≤ 0.05.

## Results

### Preparation of Blue Mussel Protein Hydrolysate

Defatted proteins derived from blue mussel (*M*. *edulis*) were hydrolyzed with alcalase, and chemical analyses showed the protein content in the freeze-dried HBMP reached to 62.92% (S2 Table). Amino acid analysis of HBMP showed the content of glutamic acid was the highest (14%), followed by aspartic acid (11.29%), arginine (8.80%) and lysine (8.13%) (S3 Table).

### Purification and Identification of the ACE Inhibitory Peptides from HBMP

**Fig 1:**
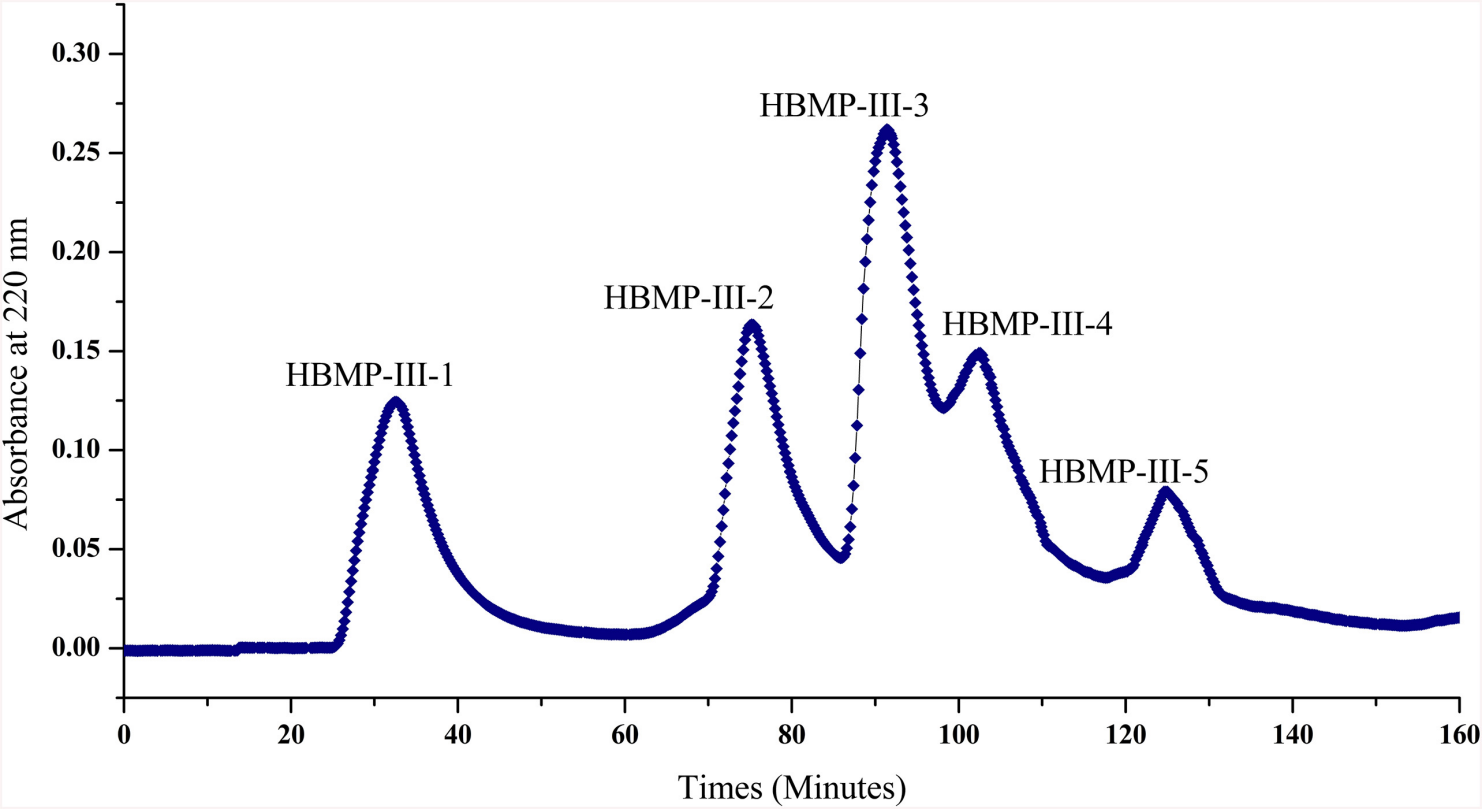
Gel filtration chromatography of HBMP-III. Low molecular weight fraction of the hydrolysate (300 mg, less than 3 kDa) was separated by the BioLogic DuoFlow medium-pressure liquid chromatography system, using a Sephadex G-15 column with distilled water at a flow rate of 1 mL/min.

Using the two MW cut-off membranes (10 and 3 kDa), three fractions were separated from HBMP and named as HBMP-I (> 10 kDa), HBMP-II (3–10 kDa), and HBMP-III (< 3 kDa), respectively. As shown in Table 1, HBMP-III showed higher ACE inhibitory activity than the other two fractions at the concentrations of 10 mg/mL. Further, HBMP-III was separated into five subfractions by sephadex G-15 chromatography (Fig 1). The third peak (HBMP-III-3) exhibited the strongest ACE inhibitory activity, although activity was widely observed among all fractions. Then HBMP-III-3 was subjected to RP-HPLC, eluted peaks with ACE inhibition were repeatedly chromatographed and three peptides were finally estimated to be Val-Trp, Leu-Gly-Trp, and Met-Val-Trp-Thr by UPLC-MS/MS system (S1 Fig). However, as these results are not compared with the UPLC-MS/MS chromatograms of standard Val-Trp, Leu-Gly-Trp, and Met-Val-Trp-Thr peptides, further studies are required to confirm amino acid sequences of the purified peptides.

**Table 1:** Molecular weight distribution of HBMP, and the ACE inhibitory activity of different fractions of HBMP.

Molecular weight	Distribution (%)	ACE Inhibitory Activity (IC_50_, mg/mL)
> 10 kDa	3.54	1.534
3–10 kDa	11.05	0.428
< 3 kDa	85.42	0.125

### Change in systolic BP of SHRs due to repeated administration of HBMP

**Fig 2:**
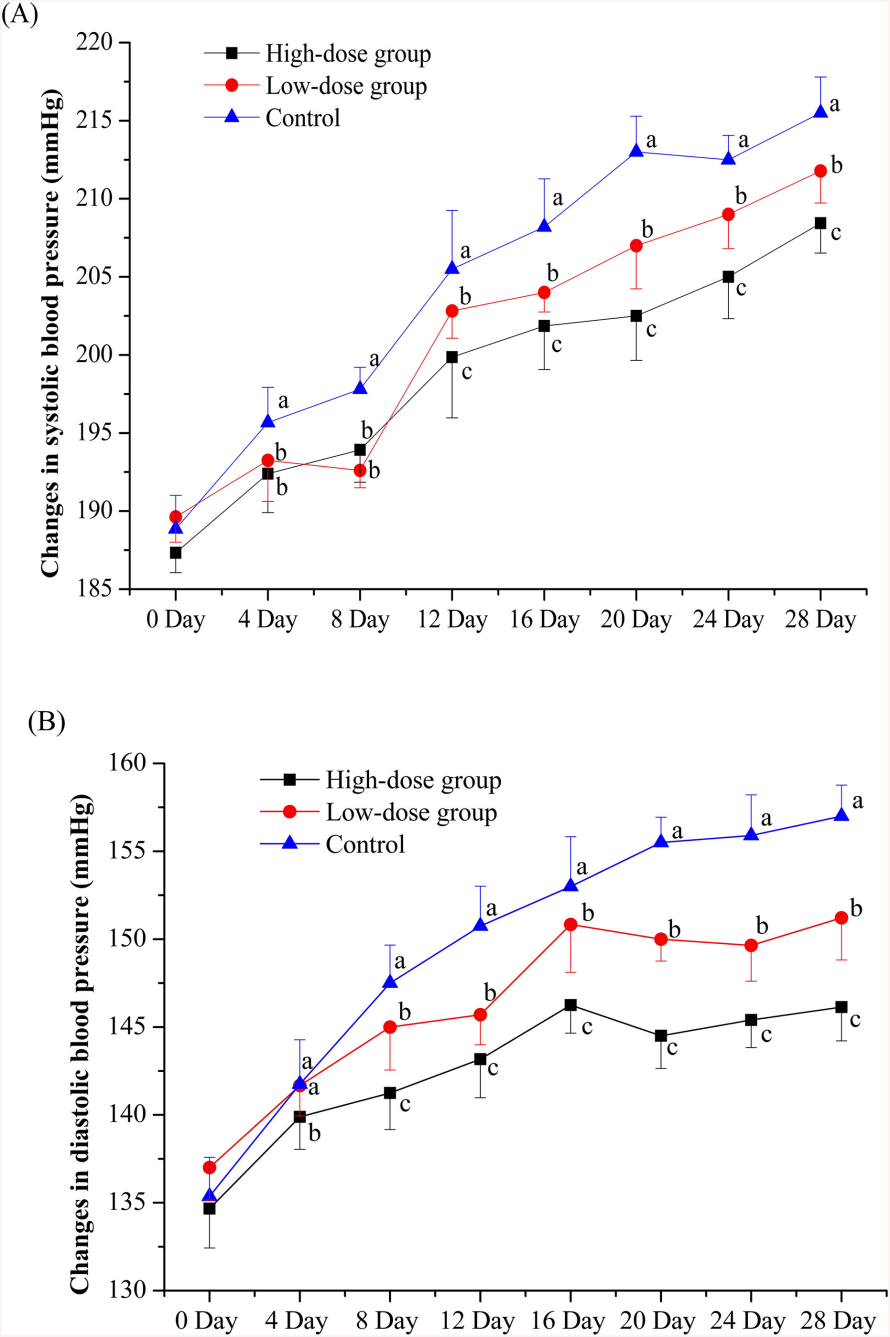
Changes in systolic blood pressure (A) and diastolic blood pressure (B) after repeated oral administration of HBMP in spontaneously hypertensive rats. Blood pressures for the HS group (■), LS group (●) and the control group (▲) were measured at 0, 4, 8, 12, 16, 20, 24 and 28 days after the start of the treatment as described in Materials and methods. Values with dissimilar lowercase letters (a-c) were significantly different, *p* value ≤ 0.05, n = 10.

According to *in vitro* ACE inhibition assays, we found the activity was widely observed among all fractions of HBMP. Thus, the crude hydrolysate including all fractions of HBMP was orally administered by gavage to SHRs. Prior to DNA microarray analyses, changes in BP of SHRs were measured on 0, 4, 8, 12, 16, 20, 24 and 28 days, at 2 hours after HBMP treatment. As the results shown in Fig 2, both the systolic BP and diastolic BP were mildly decreased in the rats of high- and low-dose groups. These results suggested HBMP could be potentially used in the prevention of hypertension. Changes of body weight of SHRs in the course of experiment was shown in S4 Table, which indicated the increases of body weight of control rats were greater than those of rats with diet supplemented with HBMP.

### Effect of HBMP on major component of RAS system in serum of SHRs

**Fig 3:**
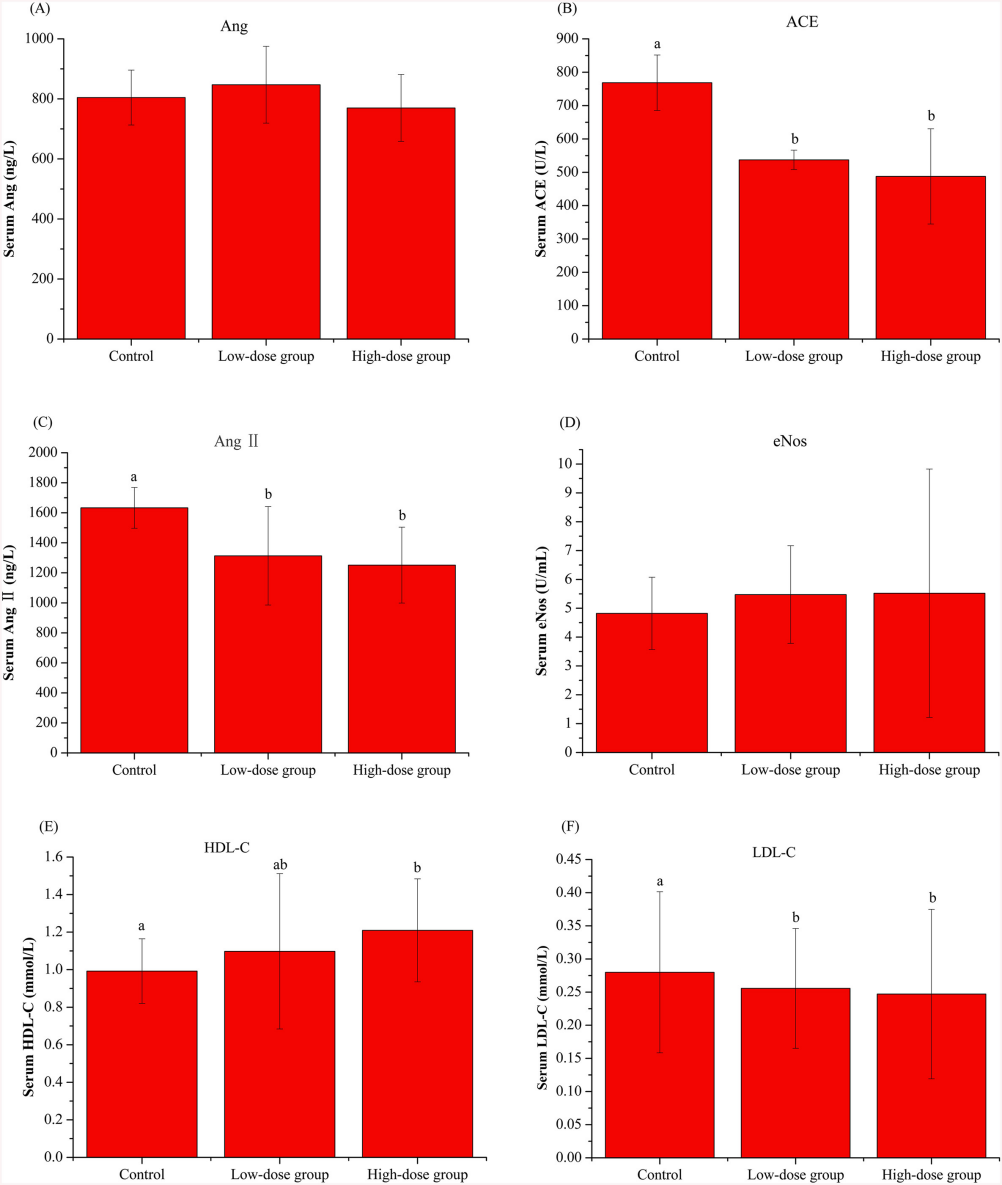
The regulative effect of HBMP on major RAS components in serum of SHRs. (A) Changes of serum Ang concentrations. (B) Changes of serum ACE activities. (C) Changes of serum AngIIconcentrations. (D) Changes of serum eNos activities. (E) Changes of serum HDL-C levels. (F) Changes of serum LDL-C levels. Values with dissimilar lowercase letters (a-c) were significantly different, *p* value ≤ 0.05, n = 10.

As the results shown in Fig 3, HBMP induced a decrease in serum ACE and AngII levels. The serum levels of Ang were not significantly differed among groups, whereas the levels of eNos were slightly elevated after administration of HBMP. When compared with the control rats, the HDL-C levels in the serum were increased whereas LDL-C levels were slightly decreased in SHRs of the high- and low-dose groups.

### Effect of HBMP on differential gene expression in kidney of SHRs

We used 44K gene chip microarray to identify differentially expressed genes in the kidney of control SHRs and SHRs treated with HBMP. When the threshold was set as 1.5, a total of 2,186 probes were found to be differentially expressed in high-dose group compared with the control rats. These probes represent 1,621 unique genes (871 were up-regulated and 750 were down-regulated) (S5 Table). Similarly, 1,921 probes (1,568 unique genes) were altered after administration of the low-dose HBMP, including 1041 up-regulated and 527 down-regulated genes compared with the control SHRs (S6 Table). These data have been deposited in NCBI's Gene Expression Omnibus and are accessible through GEO Series accession number GSE73387 (http://www.ncbi.nlm.nih.gov/geo/query/acc.cgi?acc=GSE73387).

To obtain extensive information about the *in vivo* metabolic pathways involved in the antihypertensive effect of HBMP, genes thought to be linked to pathogenesis of hypertension were firstly analyzed. As listed in Table 2, these genes could be categorized as the RAS system, vascular function, arachidonic acid system, blood coagulation system, cytokines and growth factors, fatty acid metabolism, fatty acid β-oxidation, synthesis of steroid, and insulin signaling pathway.

**Table 2:** Changes in genes thought to be linked to ACE inhibitory effect.

	Fold changes	
Genes Description	High-dose group vs. control group	Low-dose group vs. control group
**RAS system**		
angiotensin II type-1 receptor (AT-1)	1.59 (↓)	
angiotensinogen (Ang)	1.53(↓)	1.69(↓)
angiotensin I-converting enzyme (ACE)		1.73 (↓)
leucyl/cystinyl aminopeptidase	1.54(↓)	
membrane metallo-endopeptidase	1.56(↓)	2.08(↓)
**Vascular function**		
intercellular adhesion molecule 1		2.19(↓)
gap junction protein, beta 5	2.02 (↑)	2.89(↑)
tight junction protein 1	1.65(↓)	
endothelin 3	3.03(↓)	1.82(↓)
**Arachidonic acid synthesis**		
prostaglandin-endoperoxide synthase 1 (COX-1)	2.19 (↑)	3.27(↑)
prostaglandin E receptor 3	1.88 (↑)	
prostaglandin E synthase 2	1.60 (↑)	
**Blood coagulation system**		
alpha-2-macroglobulin	103.72(↑)	5.37(↑)
claudin 16	1.89(↓)	1.72(↓)
integrin, alpha 6	1.77(↓)	1.64 (↓)
laminin, gamma 2	5.12(↑)	12.39(↑)
myosin, heavy chain 7B	1.91(↓)	1.86(↓)
platelet/endothelial cell adhesion molecule 1	1.55(↓)	
selectin P ligand	1.84(↓)	2.03(↓)
vitronectin	4.37 (↓)	
versican	2.72 (↓)	11.10 (↓)
**Cytokine and growth factors**		
adiponectin	9.28 (↑)	10.36 (↑)
connective tissue growth factor	2.59 (↓)	
integrin-linked kinase	1.57 (↑)	1.51 (↑)
interleukin 1 beta	3.37 (↑)	3.82 (↑)
interleukin 17 receptor E	2.29 (↑)	5.34 (↑)
interleukin 24 (IL-24)	25.04(↑)	
interleukin enhancer binding factor 2	2.40 (↑)	2.10 (↑)
platelet derived growth factor receptor	1.57(↓)	2.22 (↓)
resistin	23.87 (↓)	21.59 (↓)
transforming growth factor, beta 2		2.51 (↓)
tumor necrosis factor, alpha-induced protein 2	1.97 (↓)	2.77 (↓)
tumor necrosis factor, alpha-induced protein 8-like	2.19 (↓)	2.13 (↓)
**Fatty acid metabolism**		
acetoacetyl-CoA synthetase	1.63 (↑)	2.13 (↑)
apolipoprotein B	1.58 (↑)	
carnitine palmitoyltransferase 1b, muscle	2.14 (↑)	3.02 (↑)
fatty acid binding protein 4, adipocyte	16.54 (↓)	9.21 (↓)
fatty acid synthase	7.66 (↑)	3.46 (↑)
peroxisome proliferator-activated receptor gamma	2.04 (↑)	3.20 (↑)
stearoyl-Coenzyme A desaturase 1 (SCD1)	19.08 (↓)	23.89 (↓)
uncoupling protein 3 (UP3)	10.68 (↑)	3.58 (↑)
**Fatty acid β-oxidation**		
acetyl-CoA acetyltransferase 1	2.29 (↑)	1.54 (↑)
peroxisome proliferator-activated receptor delta	3.42 (↑)	
**Synthesis of steroid**		
acyl-CoA binding domain containing 4	1.95 (↑)	
cytochrome P450, family 2, subfamily d	1.84 (↑)	2.32(↑)
hydroxysteroid (17-beta) dehydrogenase 8	4.41 (↑)	3.77 (↑)
hydroxy-delta-5-steroid dehydrogenase	2.56(↓)	4.44(↓)
**Insulin signaling pathway**		
insulin-like growth factor binding protein 6	4.84 (↑)	9.20 (↑)
insulin-like growth factor	1.88 (↑)	1.51 (↑)
mitogen-activated protein kinase kinase kinase	2.50 (↓)	2.02 (↓)
phosphodiesterase 1A	2.29 (↓)	1.54 (↓)
uncoupling protein 1	64.50 (↑)	17.40 (↑)

"↑" means up-regulation, and "↓" means down-regulation.

For genes associated with the RAS system, only five of them showed a tendency towards lower expression resulting from the intake of HBMP (Table 2). Gene expression of AT-1 and Ang were slightly down-regulated (by 1.59- and 1.53-fold) in SHRs of the high-dose group, as compared with the control rats. In addition, gene expression of Ang and ACE were also slightly decreased (by 1.59- and 1.53-fold) in SHRs of the low-dose group. Leucyl/cystinyl aminopeptidase, also known as vasopressinase, is a physiologically essential enzyme that cleaves peptide bonds of vasopressin sequentially from the amino terminus and thus contributes to regulation of circulating vasopressin levels [20]. Membrane metallo-endopeptidase, also known as neutral endopeptidase 24.11 (EC 3.4.24.11), usually catalyzes the degradation of atrial natriuretic peptide (ANP), whereas ANP causes vasodilation and natriuresis by direct actions that are primarily cGMP-mediated [21]. Expression levels of genes encoding these two enzymes were down-regulated after administration of HBMP in our study.

Next, genes associated with vascular function were analyzed. The most noticeable differences in this category were the down-regulation of intercellular adhesion molecule 1 gene with 2.19-fold and the up-regulation of gap junction gene with 3.97-fold in SHRs of the low-dose group. The expression of gene encoding endothelin 3 was reduced by 3.03- and 1.82-fold in SHRs of the high- and low-dose groups, respectively.

For the arachidonic acid system, the expression levels of *COX-1* gene were up-regulated after administration of HBMP, with about 2.19- and 3.27-fold. Besides these, the averaged gene expression of prostaglandin E receptor 3 and prostaglandin E synthase 2 were also slightly increased in the high-dose group than in the control rats.

Among genes associated with the blood coagulation system that were known to have an effect on BP, the expression of gene encoding alpha-2-macroglobulin showed the greatest up-regulation in the low-dose group. Whereas there were down-regulation tendencies for genes encoding cell adhesion molecules.

In terms of cytokine production, the expression levels of adiponectin and IL-24 genes showed the most significant increases. Besides, resistin gene was significantly down-regulated by 23.87- and 21.59-fold in SHRs of the high and low-dose groups, as compared with the control rats.

For fatty acid metabolism, the expression levels of genes encoding the key enzymes in fatty acid synthesis such as fatty acid synthase (FAS), fatty acid binding protein 4 (FABP4), stearoyl-Coenzyme A desaturase 1 (SCD1) and uncoupling protein 3 (UP3) were significantly altered. For β-oxidation related genes, the expression of acetyl-CoA acetyltransferase 1 and Pparδ genes were up-regulated. For the synthesis of steroid, the expression of hydroxysteroid (17-beta) dehydrogenase 8 gene was significantly up-regulated in SHRs of both the high- and low-dose groups. In addition, there were tendencies towards increasing in components of cytochrome P450. However, gene expression of hydroxy-delta-5-steroid dehydrogenase was decreased by 2.56- and 4.44-fold after administration of HBMP, in SHRs of the high- and low-dose groups, respectively.

For the insulin signaling pathway related genes, expression of uncoupling protein 1 (UP1) was most significantly elevated by 17.40- and 64.50-fold after administration of HBMP. Besides this, genes encoding insulin-like growth factor and insulin-like growth factor binding protein were increased, whereas genes encoding mitogen-activated protein kinase kinase kinase and phosphodiesterase 1A were down-regulated after administration of HBMP.

### Other up- and down-regulated genes

In our study, some genes that were seldom reported to be related with BP also showed expression changes after administration of HBMP. The most significantly up- or down-regulated genes (fold changes >10 in one or both groups) were listed in Table 3. Among them, the expression of genes encoding N-acetyltransferase 8-like (NAT8L), iron and 2-oxoglutarate dependent oxygenases domain containing 2 (OGFOD2), retinol binding protein 7 (RBP7) and 5-hydroxytryptamine receptor 2B (5-HTR_2B_) were extremely increased in SHRs after administration of HBMP. The gene expression of neuregulin 2 (NRG2), AR-β3 and thyroid hormone responsive (THR) were also significantly increased in the high- and low-dose groups, by about 20-fold. When compared with the control rats, only the expression of genes encoding ependymin related protein 1 (EPDR1), matrix metallopeptidase 7 (MMP7) and mesothelin were significantly down-regulated after intake of HBMP.

**Table 3:** Other up- and down-regulated genes detected in the kidney of SHRs after administration of HBMP.

	Fold changes	
Gene description	High-dose group vs. control group	Low-dose group vs. control group
**Up-regulated**		
N-acetyltransferase 8-like	4753.70 (↑)	7094.73(↑)
2-oxoglutarate and iron-dependent oxygenase domain containing 2	3031.43 (↑)	2140.08(↑)
retinol binding protein 7	2289.07 (↑)	3806.81(↑)
5-hydroxytryptamine (serotonin) receptor 2B, G protein-coupled	640.22(↑)	3706.31(↑)
neuregulin 2	20.00(↑)	
adrenoceptor beta 3	18.28 (↑)	14.86 (↑)
thyroid hormone responsive	11.81 (↑)	21.98(↑)
FXYD domain-containing ion transport regulator 3	7.94 (↑)	20.53(↑)
glycogen synthase 2	8.74 (↑)	19.56(↑)
branched chain amino acid transaminase 1	3.56 (↑)	16.21(↑)
apolipoprotein L3-like	5.61 (↑)	16.17(↑)
solute carrier organic anion transporter family		14.95(↑)
cytochrome c oxidase, subunit VIIIb	4.38 (↑)	14.91(↑)
annexin A8	5.98 (↑)	14.45 (↑)
6-phosphofructo-2-kinase/fructose-2,6-biphosphatase 1	7.08 (↑)	13.38 (↑)
glycoprotein (transmembrane) nmb	1.94 (↑)	12.61 (↑)
keratin 15	4.23 (↑)	12.33 (↑)
aldo-keto reductase family 1, member C-like	4.56 (↑)	11.97 (↑)
ATPase, Na+/K+ transporting, alpha 2 polypeptide	4.38 (↑)	11.25 (↑)
branched chain amino acid transaminase 1	3.56(↑)	16.21(↑)
**Down-regulated**		
ependymin related protein 1 (zebrafish)	33.90(↓)	3.76 (↓)
matrix metallopeptidase 7	4.41(↓)	110.83(↓)
mesothelin	3.03 (↓)	11.64 (↓)

"↑" means up-regulation, and "↓" means down-regulation.

### Gene ontology (GO) processes

**Fig 4:**
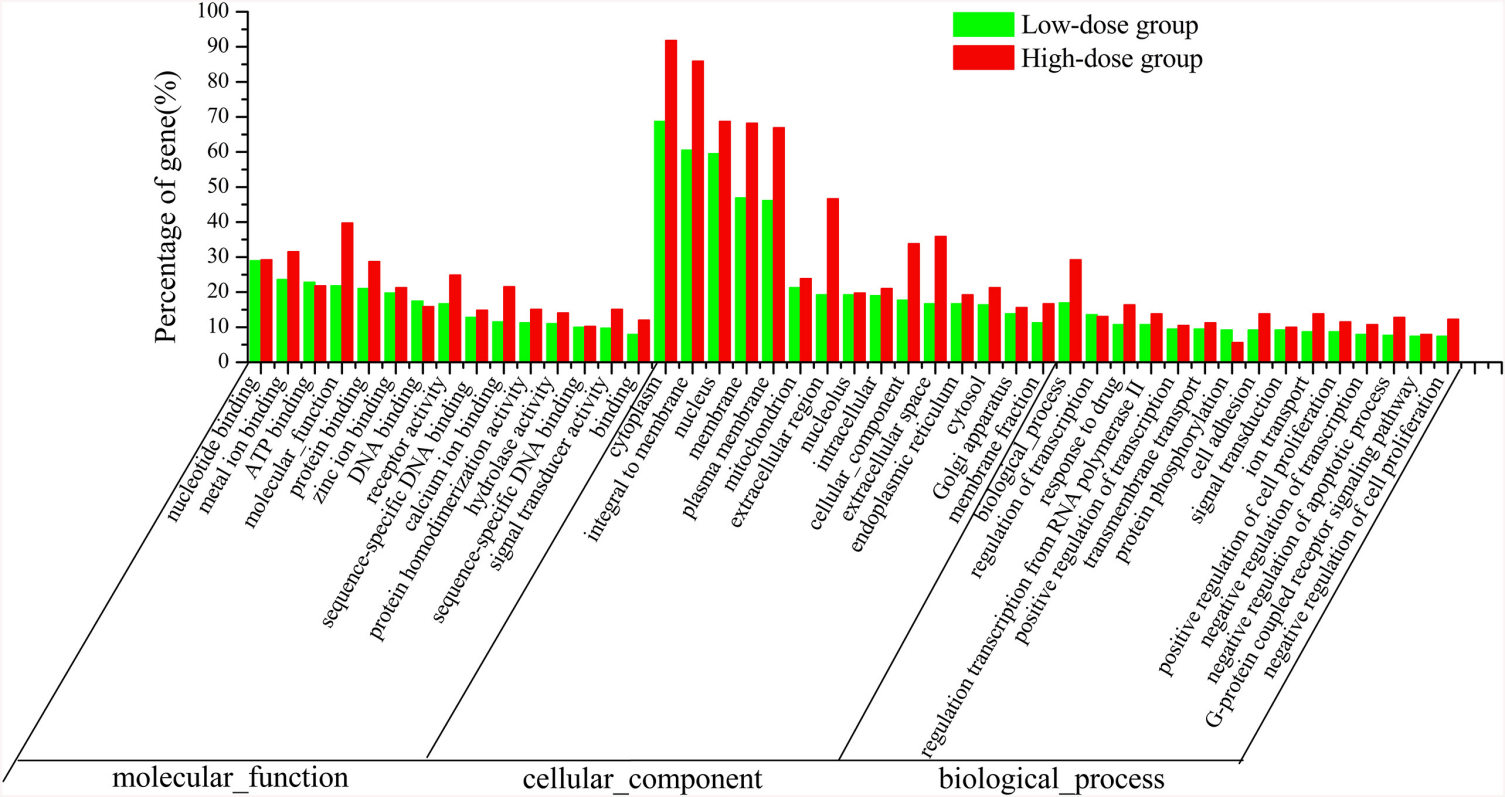
The distribution of top 15 enriched GO terms in biology process, cellular component and molecular function for the differentially expressed genes after administration of HBMP. Green bars indicate the enrichment of the GO terms for differentially expressed genes in the low-dose group when compared with the control group, and red bars indicate the enrichment of the GO terms for differentially expressed genes in the high-dose group when compared with the control rats.

As the results shown in S7 Table, the differentially expressed genes were sorted as 4548 GO terms when rats in the high-dose group were compared with the control rats. Among them, 3000 were biological process-related pathways, 473 were cellular component-related pathways, and 1075 were molecular function-related pathways. When rats in the low-dose group were compared with the control rats, the differentially expressed genes were sorted as 4607 GO terms, including 3115 biological process-related pathways, 440 cellular component-related pathways, and 1052 molecular function-related pathways (S8 Table). Both of the results indicated that the total number of genes involved in regulation of biological processes was greatest among the differently expressed genes. However, when the distribution of top 15 enriched GO terms in three pathways were analyzed, we found the frequency (number) of genes involved in cellular component such as cytoplasm, integral to membrane, nucleus, membrane, plasma membrane, and extracellular region were most enriched after HBMP treatment (Fig 4).

KEGG pathway analyses were also performed. These differentially expressed genes were identified to be enriched in metabolic pathways, pathways in cancer, focal adhesion, cell adhesion molecules and MAPK signaling pathway (S9 and S10 Tables).

### Validation of microarray data by qRT-PCR

**Fig 5:**
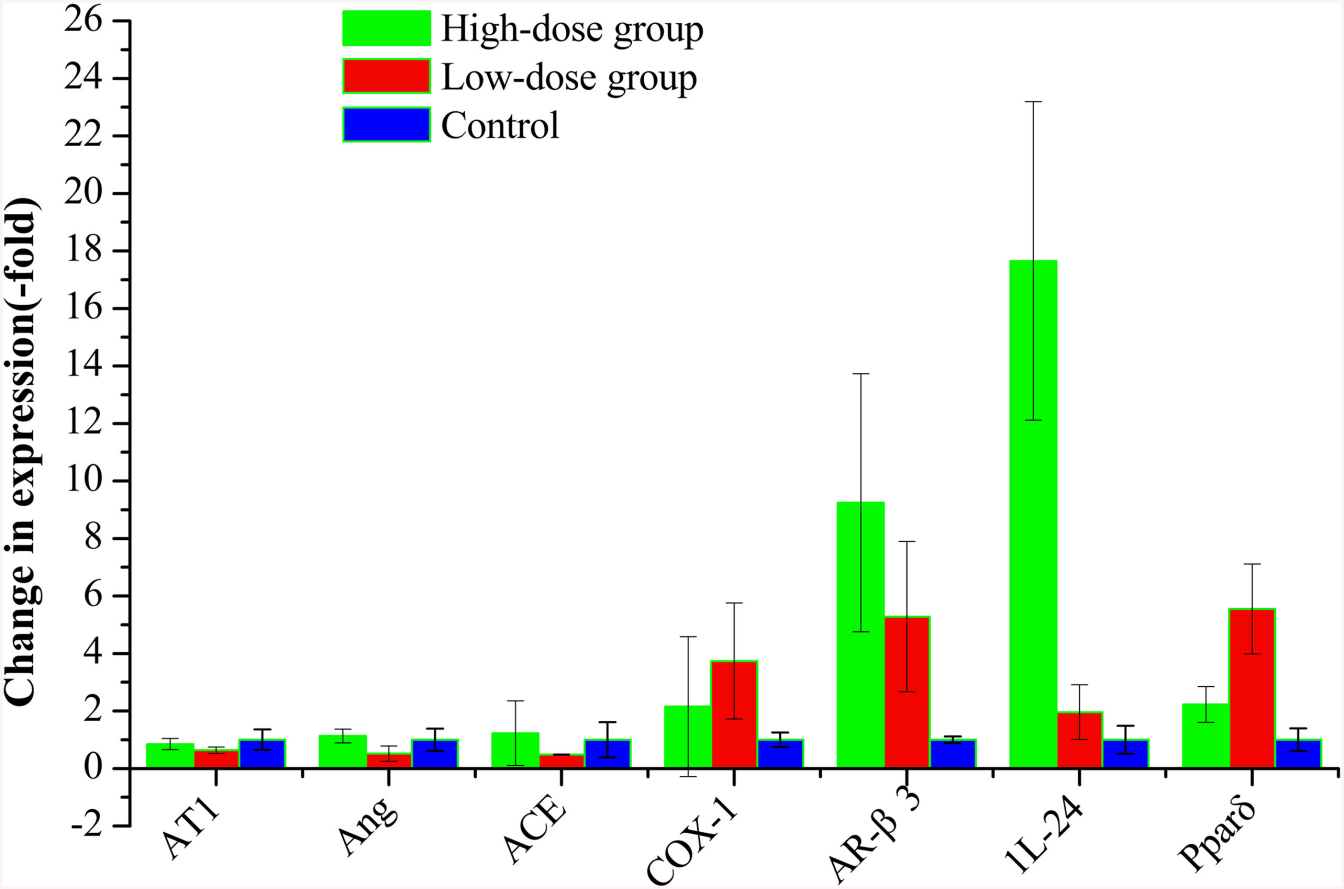
Real-time quantitative RT-PCR analyses of angiotensinogen(*Ang*), angiotensin II type-1 receptor (*AT1*), angiotensin-I-converting enzyme (*ACE*), prostaglandin-endoperoxide synthase 1 (*COX-1*), adrenoceptor β-3 *(AR-β3)*, *I*nterleukin 24 (*IL-24*) and peroxisome proliferator-activated receptor δ (*Pparδ*) genes expression in kidneys of control and HBMP-treated rats. According to the comparative method (RQ = 2^–ΔΔCt)^, the expression level of each gene was first normalized to β-actin (reference gene), and then made relative to the amount of corresponding gene in control group, representing the calibrator. All reactions of qRT-PCR were repeated three times for each sample, and vertical bars indicate standard errors.

To confirm the accuracy of the results obtained from DNA microarray analyses, the expression of several randomly selected genes were confirmed by qRT-PCR. As the results shown in Fig 5, the qRT-PCR data indicated that the expression patterns of *AR-β3* and *IL-24* were most significantly up-regulated in rats of the high-dose group, whereas the *COX-1* and *Pparδ* levels were most noticeable up-regulated in the low-dose group. These results agreed with the data obtained by microarray. However, discrepancies were also observed between the qRT-PCR and microarray data in expression patterns of *Ang* and *ACE* (Fig 5). As expected, the largest discrepancies were observed among the genes expressed at low levels, possibly because of differences in the sensitivity and specificity between qRT-PCR and microarray technology [22,23]. Besides these, the microarray data were processed by quantile algorithm, whereas qRT-PCR results were normalized by the β-actin.

## Discussion

Currently, consumer's interest in the relationship between diet and health has increased substantially. Besides their basic nutrition, more and more consumers want foods that have the potential to improve health, increase longevity and/or reduce the risk of, or delay the onset of, disease [10]. Up to date, many bioactive peptides have been discovered from various kinds of hydrolyzed food protein [24,25]. Among them, the antihypertensive peptides are receiving special attention due to the high prevalence of hypertension and its role in cardiovascular diseases (CADs). Generally, their antihypertensive effects are considered to be caused by the ACE inhibitory activities, which are often studied *in vitro*. However, the underlying mechanism is seldom studied. In this paper, we carried out gene profiling of the kidney of SHRs after repeated oral administration of HBMP, to understand the underlying mechanism of its BP lowering effect.

It is well known that regulation of BP is complex, involving a variety of molecules and intertwining metabolic pathways. Among genes previously known to be related with pathogenesis of hypertension, those associated with blood coagulation system, cytokine and growth factor, and fatty acids metabolism were noticeable changed in our study. For blood coagulation system, the genes encoding claudin molecules, integrin molecules, platelet/endothelial cell adhesion molecules, tight junction proteins, matrix metallopeptidase and extra-matrix deposition molecules were noticeable down-regulated in the kidney of SHRs feeding with HBMP. Similar results were also found by Yamaguchi et al. [26] on SHRs after VPP and IPP treatment.

For cytokines and growth factors, we found an up-regulation in secretion of adiponectin, whereas the secretion of resistin, TNF-α and TNF-β were decreased in SHRs feeding with HBMP. Many previous studies have proved that adiponectin, as the prototype of anti-inflammatory adipocytokines, is a useful biomarker related with disease progression of heart failure from hypertension in rats, and low adiponectin level in serum is associated with decreasing of cardiac diastolic function [27]. Resistin is another adipocytokine regulating carbohydrate and lipid metabolism. Zhang and his colleagues [28] reported that serum resistin concentrations in type 2 diabetes mellitus were significantly higher than those in impaired glucose tolerance and in normal glucose tolerance patients. The decreased expression levels of resistin observed in our study were in line with these previous studies, as SHRs were reported to be predisposed to insulin resistance [29].

IL-24 was known as melanoma differentiation antigen 7 exhibiting proapoptotic activity in a variety of tumor cells and belonging to the IL-10 family of cytokines [30]. Previous studies reported several anticancer functions of IL-24, including cancer-specific induction of apoptosis, cell cycle regulation, and the ability to inhibit angiogenesis [31]. However, the role of IL-24 on the pathogenesis of hypertension was seldom studied. We found the *IL-24* expression level was increased by 25.04-fold in SHRs of the high-dose group. This result was consistent with that of Lee and his colleagues, who also reported the up-regulation of *IL-24* in SHRs feeding with enalapril and nifedipine [32]. They also found that exogenous administration of IL-24 attenuated the expression of vascular inflammation- and hypertension-related genes induced by H_2_O_2_ treatment in mouse vascular smooth muscle cells [32]. These data suggested that *IL-24* might be a novel therapeutic target for hypertension. However, the expression change of *IL-24* in SHRs of the low-dose group was not observed in our study. Thus, the role of *IL-24* in BP regulation needs to be further confirmed.

For genes related to fatty acid metabolism, the most striking expression changes were observed for genes encoding FABP4, UCP3 and SCD1. FABP4 is an intracellular chaperone for free fatty acids. It acts as lipid chaperone and modulates several lipid-signaling cascades. Experimental studies showed the elevation of circulating *FABP4* level was associated with obesity, insulin resistance, diabetes mellitus, hypertension, cardiac dysfunction, atherosclerosis, and CAD events [33]. Whereas reduction of *FABP4* expression leaded to a decrease in CAD events. *UCP3* encodes a member of the mitochondrial anion carrier superfamily of proteins uncoupling mitochondrial respiration. Previous studies showed that *UCP3* over-expression (roughly 3-fold) in mice muscle increased spontaneous activity (about 40%) and energy expenditure (5–10%), whereas decreased oxidative stress (15–20%) [34]. Hellsten et al. [35] also reported that UCP3 regulated reactive oxygen species levels and cell survival during hypoxia in human skeletal muscle. In our study, the down-regulation of *FABP4* and up-regulation of *UCP3* levels in SHRs treated with HBMP seemed consistent with these previous studies, suggesting both of them were contributed to BP reduction. SCD1 plays a critical role in lipid metabolism, by converting saturated fatty acids to monounsaturated fatty acids. Several studies have confirmed that SCD1 promoted both steatosis and hypertriglyceridemia, and inhibition of *SCD1* expression was extremely effective in preventing diet-induced obesity, hepatic steatosis, and insulin resistance [31,36,37]. However, it was also reported that *SCD1* inhibition or deletion did not always result in lower plasma triglyceride. Brown et al. [38] suggested that the background strain of experimental mice and diet should be taken into consideration when examining the effect of *SCD1*. We found the expression of *SCD1* were decreased by 19.08- and 23.89-fold in rats with diet supplemented with HBMP. These results suggest that *SCD1* is a noticeable factor related with BP reduction of SHRs.

Except those genes listed in Table 2 the expression of several genes which were seldom reported to be related with hypertension also showed significant alterations. NAT8L catalyzes the formation of *N*-acetylaspartate from acetyl-CoA and aspartate [39]. *N*-acetylaspartate then acts as a carrier and delivers the acetate moiety for synthesis of acetyl-CoA that is further used for fatty acid generation. Pessentheiner et al. [39] found that *NAT8L* was highly expressed in adipose tissues of rat and human, and stable over-expression of *NAT8L* in immortalized brown adipogenic cells strongly increased glucose incorporation into neutral lipids, accompanied by increased lipolysis, indicating an accelerated lipid turnover. These results are in consistent with our observation, indicate that oral administration of HBMP can accelerate lipid turnover and increase energy expenditure in SHRs.

In plants and microorganisms, OGFOD2 catalyze an extraordinarily wide range of reactions including desaturations, oxidative cyclisation, rearrangements and halogenations [40]. However, in animals including humans, to date the identified reactions catalyzed by OGFOD2 are limited to the hydroxylation of C-H bonds and N-methyl group demethylation, via C-hydroxylation, followed by the fragmentation of a hemiaminal intermediate [40]. Although these reactions are ‘simple’ hydroxylations/N-methyl demethylations, recent studies have revealed they are involved in physiologically important processes including hypoxic sensing, fatty acid metabolism, DNA repair and epigenetic regulation [41,42]. It is well known that the etiopathogenesis of hypertension is multifactorial, and many studies demonstrate that epigenetic events (including DNA methylation) are very important for the development of essential hypertension [43,44]. For example, Smolarek et al. [45] found levels of 5mC in the DNA of patients suffering from essential hypertension were lower than in healthy people and they correlated with the stage of hypertension. In our study, the expression levels of gene encoding OGFOD2 were striking increased by 3031.43- and 2140.08-fold in SHRs of the high- and low-dose groups, thus we speculated the levels of DNA methylation might changed in SHRs after repeated oral administration of HBMP. However, further study is needed to confirm such speculation.

RBP7 is a PPARγ target protein highly expressed in endothelium. Hu and his colleagues [46] reported that *RBP7* might normally mediate protective effects of PPARγ and that deletion of *RBP7* might augment Ang II-induced endothelial dysfunction in mice, implying *RBP7* was involved in BP regulation. The *RBP7* levels were increased by 2289.07- and 3806.81-fold in SHRs of the high- and low-dose groups, which might indirectly contribute to BP reduction.

5-HT is a hormone/neurotransmitter, which exerts its biological effects primarily through activation of receptors in cell membrane. Seven major families of 5-HT receptors and subtypes exist, and most of them are heptahelical receptors coupled to G proteins [47]. *In vitro*, 5-HT was observed as a vasoconstrictor for a long time. However, Fregly et al. [48] demonstrated that 5-HT, given *in vivo*, reduced BP. Therefore the role of 5-HT and its receptors in BP regulation is controversial currently [47]. Several independent laboratories demonstrated that administration of the 5-HT either acutely or more chronically (12 days), reduced BP of the SHRs, Sprague-Dawley rats, Dahl salt sensitive rats and DOCA-salt hypertensive rats [49–51]. Our data showed the expression of 5-HTR_2B_ was significantly increased in HBMP treated rats, which seemed consistent with the findings of SHRs in previous reports. Yet the underlying mechanisms were unclear as the plasma levels of 5-HT were not studied in our study.

NRG2 is a member of the epidermal growth factor family, which binds directly to epidermal growth factor receptor 3 (ErbB3) and ErbB4 [52]. As ErbB signaling has been implicated in angiogenesis and endothelial cell proliferation, NRG2 might also have relevant role on the development of hypertension. In facts, Matsukawa et al. [53] reported that NRG/ErbB acting as an antihypertensive system, and inhibition of ErbB2 expression leaded to hypertension, at least in part, by reducing nitric oxide synthesis and inhibiting γ-aminobutyric acid activity. The up-regulation of *NRG2* levels in kidney of SHRs treated with HBMP observed in our study seemed consistent with these reports, indicating NRG/ErbB signal pathway were involved in the antihypertensive effect of HBMP.

AR-β3 is an important regulator of the cardiovascular system and of endothelial cell function in particular. Perros et al. [54] demonstrated that activation of the AR-β3 by nebivolol, a drug with β-2,3-adrenegic agonist and β-1-antagonist properties, could enhance endothelial nitric oxide production and decrease the generation of reactive oxygen species, leading to pulmonary vasodilation, and attenuated vascular remodeling. Thyroid hormone is another key regulator of lipid metabolism besides insulin and glucose. Ortega et al. [55] reported that *THR* was directly associated with adipogenesis in human adipocytes, but inversely related to obesity and omental fat. In our study, the elevated expression levels of *AR-β3* and *THR* in HBMP-treated SHRs indicated that they might also contribute to BP reduction.

When compared with the control rats, only the expression of *EPDR1* and *MMP7* were significantly down-regulated with fold changes bigger than 20 in HBMP-treated SHRs. EPDR1 is an extracellular glycoprotein, which was postulated to be involved in intracellular signaling [56]. The down-regulation of *EPDR1* in our study indicated the altered signaling pathway after intake of HBMP. The *MMP7* levels were decreased by 4.41- and 110.83-fold in SHRs of the high- and low-dose groups, respectively. These were consistent with previous reports, as MMP7 and other members in the matrix metallopeptidase family were reported to play important roles in extracellular matrix turnover, cancer cell migration, cell growth, inflammation, and angiogenesis [57]. Wang et al. [58] suggested that posttranscriptional activation of MMP7 was required in vasoconstrictors induced hypertension, and blocking MMP7 expression could be valuable for attenuating hypertension and preventing the development of cardiac hypertrophy.

The most interesting finding in our study was that we found HBMP employed multiple mechanisms in exerting its antihypertensive effect other than ACE inhibition, showed by the slightly changes of ACE levels among different group of SHRs, according to the results of ELISA, microarray and qRT-PCR assays. On the other hand, genes that were seldom reported to be related with hypertension showed extremely expression changes, such as the *NAT8L*, *OGFOD2*, *RBP7*, *5-HTR*_*2B*_, *AR-β3* and *EPDR1*. Based on their known functions, these data may aid in the delineation of the molecular mechanisms that underlie the potential antihypertensive activities of HBMP.

There were several limitations of our study. Firstly, part of the whole kidney was used to look at the HBMP effect on genetic expression. The kidney tissue consists of a mixture of different cell types. Thus, we couldn't completely exclude that some genetic differences might be due to the different composition of cell types that were presented in different samples. Secondly, microarray analyses for other organs such as the lung, aorta and liver would be helpful for full understanding the *in vivo* actions of HBMP. Thirdly, the short experimental period was clearly another limitation of our study. If the duration of the treatment were expanded, the inhibition of BP increase in the groups receiving HBMP might reach a significant levels.

Taken together, we analyzed the chemical component of HBMP and purified the *in vitro* ACE inhibitory peptides from the hydrolysate, and reported the overall gene expression profiles from kidney of SHRs orally treated with HBMP for the first time. Our results suggest that dietary intake of HBMP had mild BP lowering effect, and the data from DNA microarray analyses clearly showed between-group differences in up- and down-regulated genes expression, including several genes that had not been implicated in pathogenesis of hypertension. Furthermore, these data suggested that HBMP employed multiple mechanisms in exerting its physiological antihypertensive effect other than ACE inhibition. Although the obtained results were different from the traditional understanding of active peptides, the differentially expressed genes identified here could help our understanding and potential utilization of HBMP in the treatment or prevention of hypertension.

## Supporting Information

S1 Fig UPLC/MS/MS chromatograms of peptide Val-Trp (A), Leu-Gly-Trp (B), and Met-Val-Trp-Thr (C). (DOCX)

S1 Table Primers used for qRT-PCR in this study. (XLS)

S2 Table Chemical composition of raw muscle and protein hydrolysates of blue mussel, expressed as percentage of dry matter. (XLS)

S3 Table The amino acids composition of the protein hydrolysates of blue mussel. (XLS)

S4 Table Body weights of SHRs during the experiment period. (XLS)

S5 Table Profile of differently expressed genes in the kidney of SHRs between control and high-dose groups. (XLS)

S6 Table Profile of differently expressed genes in the kidney of SHRs between control and low-dose groups. (XLS)

S7 Table GO analyses of the differently expressed genes in the kidney of SHRs between control and high-dose groups. (XLS)

S8 Table GO analyses of the differently expressed genes in the kidney of SHRs between control and low-dose groups. (XLS)

S9 Table Significantly enriched KEGG pathways in the differently expressed genes between (XLS)

S10 Table Significantly enriched KEGG pathways in the differently expressed genes between control and low-dose SHR groups. (XLS)
