# Supplementary material for: Alteration of Gene Expression Profile in Kidney of Spontaneously Hypertensive Rats Treated with Protein Hydrolysate of Blue Mussel (Mytilus edulis) by DNA Microarray Analysis
Source: PLoS One. 2015 Oct 30;10(10):e0142016. doi: 10.1371/journal.pone.0142016 (PMC4627735; doi:10.1371/journal.pone.0142016)
Supplement: S1 Fig — (DOCX) [file pone.0142016.s001.docx]

(A)

(B)

(C)

**S1 Figure.** **UPLC/MS/MS chromatograms of peptide Val-Trp (A), Leu-Gly-Trp (B), and Met-Val-Trp-Thr (C).**
